# Selecting the Best: Evolutionary Engineering of Chemical Production in Microbes

**DOI:** 10.3390/genes9050249

**Published:** 2018-05-11

**Authors:** Denis Shepelin, Anne Sofie Lærke Hansen, Rebecca Lennen, Hao Luo, Markus J. Herrgård

**Affiliations:** The Novo Nordisk Foundation Center for Biosustainability, Technical University of Denmark, 2800 Kongens Lyngby, Denmark; denshe@biosustain.dtu.dk (D.S.); aslh@biosustain.dtu.dk (A.S.L.H.); rlen@biosustain.dtu.dk (R.L.); hluo@biosustain.dtu.dk (H.L.)

**Keywords:** evolutionary engineering, ALE, metabolic engineering, bioproduction, genetic engineering, growth coupling

## Abstract

Microbial cell factories have proven to be an economical means of production for many bulk, specialty, and fine chemical products. However, we still lack both a holistic understanding of organism physiology and the ability to predictively tune enzyme activities in vivo, thus slowing down rational engineering of industrially relevant strains. An alternative concept to rational engineering is to use evolution as the driving force to select for desired changes, an approach often described as evolutionary engineering. In evolutionary engineering, in vivo selections for a desired phenotype are combined with either generation of spontaneous mutations or some form of targeted or random mutagenesis. Evolutionary engineering has been used to successfully engineer easily selectable phenotypes, such as utilization of a suboptimal nutrient source or tolerance to inhibitory substrates or products. In this review, we focus primarily on a more challenging problem—the use of evolutionary engineering for improving the production of chemicals in microbes directly. We describe recent developments in evolutionary engineering strategies, in general, and discuss, in detail, case studies where production of a chemical has been successfully achieved through evolutionary engineering by coupling production to cellular growth.

## 1. Introduction

The idea of using microbes as cell factories for chemicals has a long history and is well established now as an industrial activity [1]. Biobased production is a promising route to a more sustainable chemical industry, although there are lots of pitfalls and challenges on the way to converting chemical production processes to biochemical ones. Despite major success stories, such as commercial-scale production of the bulk chemical 1,4-butanediol (1,4-BDO) [2] or the pharmaceutical precursor artemisinic acid [3], the development of economically viable production strains and processes is still time consuming and prone to failures [4]. One of the most important factors slowing down strain and process development is lack of comprehensive understanding of the host organisms, even in cases where well-studied bacterial and fungal hosts are used. Traditional engineering approaches require substantial quantitative knowledge of the system that is being engineered, and the ability to reliably predict system response to genetic or environmental manipulations. Even though there has been notable progress in characterizing and predicting cellular functions, there are still many uncertainties, especially when the cells are subjected to multiple simultaneous genetic or environmental changes [5,6]. 

One way of dealing with the complexity of engineering living cells is to pass the strain optimization task to nature itself. Using evolution as driving force we can achieve phenotypic improvements that are hard to obtain by rational genotype-driven engineering, as will be highlighted in this review [7]. Evolution in the laboratory was first researched by Louis Pasteur and Robert Koch, and the first published adaptive laboratory evolution (ALE) experiments were performed by William Dallinger more than hundred years ago [8]. From the 1960s and onwards, there have been increasing efforts to employ ALE for engineering of strains (also known as evolutionary engineering) to achieve desired functions [9,10]. Evolutionary engineering possesses clear benefits over rational approaches, including broad applicability to different microbial hosts [11], ease of practical implementation, ability to discover new mechanisms that are non-intuitive, and the ability to guarantee at least some improvement in industrially relevant phenotypes [9]. Recent developments in DNA sequencing and omics technologies allow for high throughput assessment of evolved populations and isolates at the molecular level [12,13]. This enables using ALE experiments as a tool to discover new biological mechanisms, such as previously uncharacterized enzyme activities that can further be used as part of rational strain development activities.

**Figure 1:**
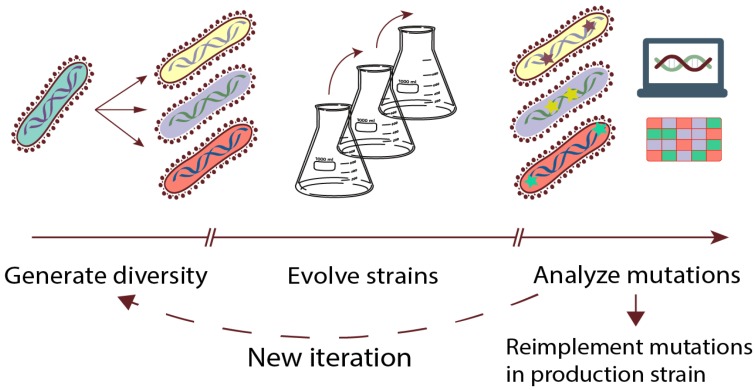
Adaptive laboratory evolution (ALE) workflow. Experiment starts by generating an initial population with or without genotypic diversification, followed by evolution for a desired time, and finally, analysis of populations and/or isolates for beneficial mutations. ALE can be performed sequentially with a starting population from a previous run or as a single run experiment. After ALE, the resulting isolates can be used directly as they are, but often mutations are reimplemented in a clean production strain.

The workflow of a typical ALE experiment is briefly outlined in Figure 1, by dividing it into three phases: (1) initial diversity generation of starting population(s), (2) passage of populations under selective conditions to enrich for superior variants, and (3) analysis of the population or single isolates to identify causal mutations. These mutations can be further implemented in clean background strains, which can be followed by new iterations of the ALE experiment in an attempt to obtain additional beneficial mutations. Recent advances in automation technologies have allowed for automating all phases of the workflow, which increases the throughput of ALE experiments significantly compared to traditional labor-intensive approaches [14]. 

The success of an ALE experiment depends on the design of the selection regime, and designing the right selection for a given application is arguably the most critical and challenging part of the experiment. In practice, most ALE experiments select for increased growth, as growth can be measured easily and selected for in high throughput. Selection based on growth rate also makes it harder for the cells to escape from the selection system and stop production of a desired product [15,16].

One of the most popular applications for evolutionary engineering is to evolve strains to tolerate adverse environmental conditions or to utilize alternative feedstocks [9,11,17]. The popularity of performing such studies is explained by the comparative simplicity of designing the selective pressure. Cells are simply exposed to a suboptimal environment, such as the presence of a toxic compound, and it is quite unlikely that cells can easily escape such selection and optimize for a different phenotype than is intended for. Examples of these types of evolutionary engineering studies include evolution for thermotolerance [18], ability to tolerate ionic liquids used in biomass hydrolysis [19], or for the ability to utilize efficiently suboptimal carbon sources, such as glycerol [20]. 

**Figure 2:**
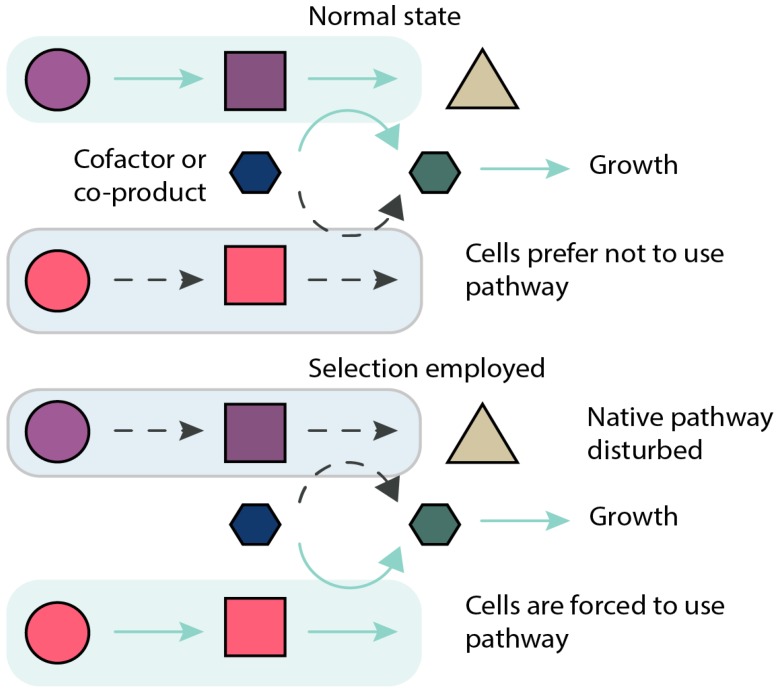
Principle of growth-coupled design. Metabolic network presented in two states: in its normal wild type state, and with the selection regime implemented. In the normal state, cells use their natural pathways for production of metabolites (green box and arrows), and as a side product, produce or recycle e.g., cofactors required for growth (green hexagon); the target pathway of interest is inactive in this state (blue box, dotted arrows). In the selection regime, the native pathway for production or recycling of essential metabolite or cofactor is compromised (blue box, dotted arrows) making the pathway of interest the sole source of essential metabolite production or cofactor recycling (dotted arrows become solid green). This couples the target pathway to cellular growth stoichiometrically.

In contrast to evolutionary engineering for tolerance or utilization, evolutionary engineering for increased production of a chemical is more challenging. This is because in most cases, production of a target chemical imposes an additional burden on cells and reduces growth [21]. In specific cases, linking production of a target chemical to growth (also known as growth coupling—Figure 2) can be achieved through manipulation of native metabolism, introduction of heterologous reactions to enforce coupling, or by changing growth conditions. In this review, we will focus on the uses of evolutionary engineering for enhancing production of chemicals through different growth-coupling strategies. We describe the full workflow of the evolutionary engineering approach, including in silico design of growth-coupling strategies, approaches to speed up evolution by creating genetic diversity, practical implementation of laboratory evolution in vivo, and the use of resequencing and other omics approaches to understand the genetic changes that occur in the evolutionary process. We conclude by highlighting some of the most prominent experimental results for evolutionary engineering of chemical production and discuss challenges of this approach.

## 2. Theoretical Frameworks for Coupling Target Metabolite Production to Growth

Genome-scale metabolic models provide a convenient framework for in silico design of strategies for coupling production of a desired chemical compound to growth. These models represent cellular metabolism in a computationally tractable form that allows linking genetic manipulations (e.g., deletions of metabolic genes) to phenotypic outcomes (e.g., production of a metabolite) [22,23]. One of the early computational tools that enabled prediction of growth-coupled production designs is the algorithm OptKnock, developed by Burgard et al. [24]. OptKnock can be applied to genome-scale metabolic models to predict optimal genetic knockouts favoring cell growth, and synthesis of the desired product based on a concept that a drain towards growth resources (i.e., carbon, redox potential, and energy) must be accompanied, due to stoichiometry, by the production of a desired product. Many other algorithms for predicting growth-coupled designs have been proposed that either speed up the computations or allow additional genetic manipulations, such as overexpression or introduction of heterologous pathways [25]. Examples of recent methods for designing growth coupling are “SelFi” [26], which allows introducing new pathways for consumption of unusual substrates to find additional growth-coupling strategies. 

Growth coupling has been demonstrated, in silico, to be possible for almost any metabolite in *Escherichia coli* and *Saccharomyces cerevisiae* [27,28]. Despite the fact that the production of almost any native metabolite could, in principle, be coupled to cell growth, most of the in silico designs are difficult to implement in practice, due to the extensive genetic manipulations needed. Furthermore, the actual growth rates for these designs can be too low for an ALE experiment, and the production rates of a target product may also be too low to be of practical value. Growth-coupled designs can be used without employing evolutionary engineering if one can rationally implement the necessary changes, such as balancing heterologous gene expression with native pathways to enable optimal coupling. Examples of such experimentally validated growth-coupled designs include coupling isobutanol production to growth through redox balance [29] (design was further improved in [30]), and the production of itaconic acid from glucose, with a product yield of 0.68 mol/(mol glucose) [31].

## 3. Creating Diversity: From Gene Level to Population Level

Evolution relies on population heterogeneity and mutations, and thus, for ALE generation of initial diversity in the population, can be beneficial, even if it is not strictly necessary. ALE can be performed without any prior mutagenesis or introduction of targeted genetic variation, but the natural mutation rate can be too low, and in cases where, for example, a heterologous enzyme needs to be optimized, “off-target” mutations outside the enzyme sequence can occur. These off-target mutations can, themselves, have value in identification of new biological mechanisms, but they can also represent escapes from the desired selection pressure (e.g., coupling production to growth).

**Figure 3:**
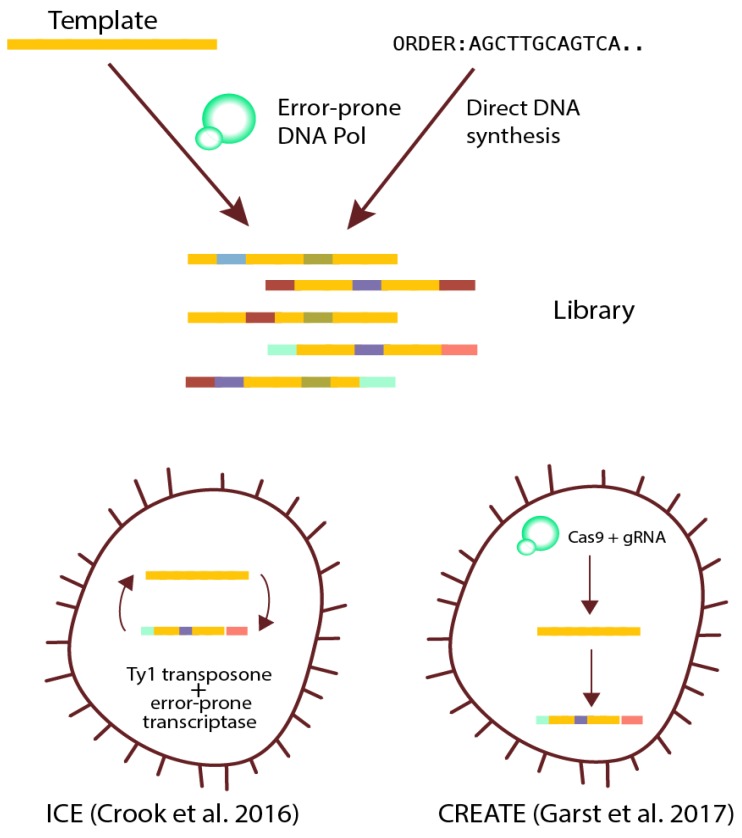
Examples of strategies for creating diversity in populations. Scheme of modern library generation techniques. Libraries can be generated via methods yielding a fixed set of variants before the ALE experiment, either at a single gene or whole genome level. Diversity can also be created by employing methods for continuous generation of genetic variants during the ALE experiment. DNA Pol: DNA polymerase; ICE: in vivo continuous evolution; CREATE: CRISPR-enabled trackable genome engineering.

Genetic diversity can be created in a variety of ways, ranging from completely untargeted genome-wide mutagenesis methods using, e.g., ultraviolet (UV) light, to fully targeted approaches, where specific libraries targeting specific positions in a gene or genome are used—Figure 3. One of the popular ways to increase diversity in a semi-targeted manner is to use random mutagenesis, such as error-prone PCR (epPCR) to generate libraries of selected genes in vitro [32]. This PCR-based method offers a rapid and economically viable way to generate a gene library using error-prone DNA polymerases in a semi-controllable manner. However, error-prone DNA polymerases can often favor certain types of mutations, thus, the mutational landscape of epPCR libraries tends to be biased, ultimately diminishing the effective size of the library collection. This can lead to poor coverage of some sites, due to their location in the sequence. These biases can be overcome by changing the proportion of dNTPs used for the reaction, or by switching DNA polymerase, with a different bias. Whole cell mutants after UV light exposure or mutator strains can be used as well, but these approaches are less popular, due to mutation biases and uncontrollable mutation rates.

If one has a reasonable idea which regions within a genome or gene are relevant for the phenotype of interest, targeted diversity generation using modern synthetic biology techniques can be used to overcome the bias and coverage issues inherent in random mutagenesis approaches. With the advent of new technologies of DNA synthesis and rational protein and regulatory element design, it is now possible to create smart gene libraries by targeting specific residues in proteins or regulatory elements, such as promoters, using gene synthesis. Recent studies show that synthetic gene libraries have much more variants compared to traditional saturation mutagenesis (97% and 50% of theoretical sequence pool, accordingly) [33]. Synthesis companies have the ability to deliver exactly defined combinations of mutations in any site, allowing generation of smart and efficient libraries. Recently emerged systems based on CRISPR (Clustered Regularly Interspaced Short Palindromic Repeats) have also been used to create similar targeted diversity at the whole genome level. One example is the CRISPR-enabled trackable genome engineering (CREATE) method [34,35], that enables high throughput mutagenesis of bacterial and yeast cells, in vivo, at multiple loci in parallel.

The methods described above allow creating diversity prior to ALE experiment, and not during the experiment. Traditionally, increasing genetic diversity during an ALE experiment has only been possible by using biased and untargeted approaches, such as the use of mutator strains or chemical mutagens. These approaches introduce severe mutational biases, and also increase the numbers of mutations significantly, which can make further analysis of evolved strains difficult. Recently, continuous evolution methods where targeted diversity is created during ALE have been developed. An example is the in vivo continuous evolution (ICE) method from the Alper lab [36], which is a transposon-based method. Employing native retrotransposable element Ty1, combined with error-prone reverse transcriptase of said element, the group were able to achieve both good coverage and efficiency of mutations. Phage-assisted continuous evolution (PACE) [37] is a phage-based method for continuous directed evolution of various proteins. The main idea of the method is to couple growth of M13 bacteriophage to the activity of a protein of interest. Main selection is targeted to phage growth, so the variety of processes that it can be coupled to is limited to the molecular life cycle of phage. This technique has been applied to engineer RNA polymerases [38], aminoacyl-tRNA synthetases [39], and even toxins [40]. For more details, we refer to recent reviews [41,42]. 

## 4. Practical Implementation of Adaptive Laboratory Evolution Experiments

After implementation of the strain design needed for the desired selection and the potential generation of initial genetic diversity, the actual ALE experiment to improve the desired phenotype can be initiated. ALE experiments for evolutionary engineering applications are usually performed in two main ways—using batch cultures with serial passages, or in chemostats. Serial passaging is simple, and the most commonly applied technique, due to several reasons. First, the manipulations needed for passaging are simple, and do not require specialized equipment. Second, these simple manipulations can be easily automated, and thus scaled up to hundreds of simultaneous experiments. There are also downsides to serial passaging. Variability of selection during the growth curve makes it hard to keep the selection pressure constant, and selection may only act on decreasing lag phase, and not actually increasing the growth rate [16]. This would be especially harmful with growth-coupled strain designs where it is essential that actual growth rate improvements are achieved in order to improve production of the target metabolite.

Chemostats make microbial culture grow at a steady state by simultaneously removing part of the culture and adding fresh medium [43]. The medium should contain some limiting factor, such as a phosphate, nitrogen, or a carbon source. The nutrient limitation creates a steady-state culture, which allows for keeping a constant selection pressure on cells. Chemostat cultures are more challenging to implement than serial batch cultures, and also, chemostats are harder to fully automate, due to issues such as contamination. Turbidostats are very close to chemostats in the way that cells are continuously removed, but there are no nutrient limitations imposed to cells. That makes turbidostats a very promising tool, combining lack of a nutrient limitation with steady-state condition, especially with the growing amount of proposed low-cost turbidostat designs, which should be affordable for many labs [44]. Experimental platforms for ALE are reviewed in greater detail in [11,45]. 

In addition to cultivation regime, one has to also determine the duration of the ALE experiment to be performed. This depends on factors such as whether initial diversity in the population has been created, how large an improvement in the target phenotype is desired, as well as practical considerations, such as cost of the experiment, in terms of human labor and supplies. The duration of an ALE experiment can be estimated based on the number of acquired mutations per generation or cumulative cell divisions [46]. The final parameter that needs to be determined for an ALE experiment is the number of independently evolved populations to be used. With the increased usage of automated ALE platforms, most studies now include several replicate populations for each initial condition, which allows for more comprehensive study of the mutational landscapes and increases the chance of identifying truly improved enzymes or strains.

## 5. Deciphering Genetic Basis for Evolved Phenotypes

The evolutionary engineering approach requires detailed analysis of evolved populations and isolates, in order connect evolutionary engineering with targeted metabolic engineering strategies. Genotyping is required to both identify causal beneficial mutations and/or uncover selection escapers, and is a crucial step in the successful implementation of selection systems for strain construction towards chemical production. Historically, the identification, characterization, and reimplementation of mutations have been laborious processes, but simultaneous advances in the DNA sequencing and other omics technologies [47], together with the expansion of the synthetic biology toolbox [48] in recent years, have made it possible to rapidly determine the causal role of mutations.

DNA sequencing can now be routinely, reliably, and cost effectively used to determine genetic variants present in evolved populations and isolates [49]. While it is now possible to identify all genetic variants, it can be hard to infer the effect of many mutations on regulation and/or metabolism. This requires using additional omics technologies, such as transcriptomics, proteomics, fluxomics, and metabolomics to provide an insight into the regulatory and/or metabolic consequences of genetic changes [50].

Evolved isolates from an evolutionary engineering study can, in principle, be used directly for production applications. However, more often, the beneficial mutations identified by evolutionary engineering are reimplemented into clean production background strains that lack the genetic changes needed for growth-coupled selections. Numerous techniques for reintroducing the mutations in a targeted manner, into either the host genome or plasmids, exist today. Due to their well-earned popularity, the expanded family of CRISPR-based methods allows for introduction of genetic changes into the genome or plasmids of almost any host organism of industrial relevance nowadays [51,52].

## 6. Case Studies of Experimental Growth-Coupling Strategies

Despite the existence of multiple computational methods for developing growth-coupled strain designs, the practical applications of growth coupling are still not common, due to difficulties in implementing the in silico designs in vivo. Here, we will describe examples of applications of growth coupling to production of chemicals, either by evolutionary engineering of whole organisms, or by engineering of enzymes that can be used for biocatalytic conversions of substrates to valuable products. The examples covered in this review are summarized in Table 1. 

**Figure 4:**
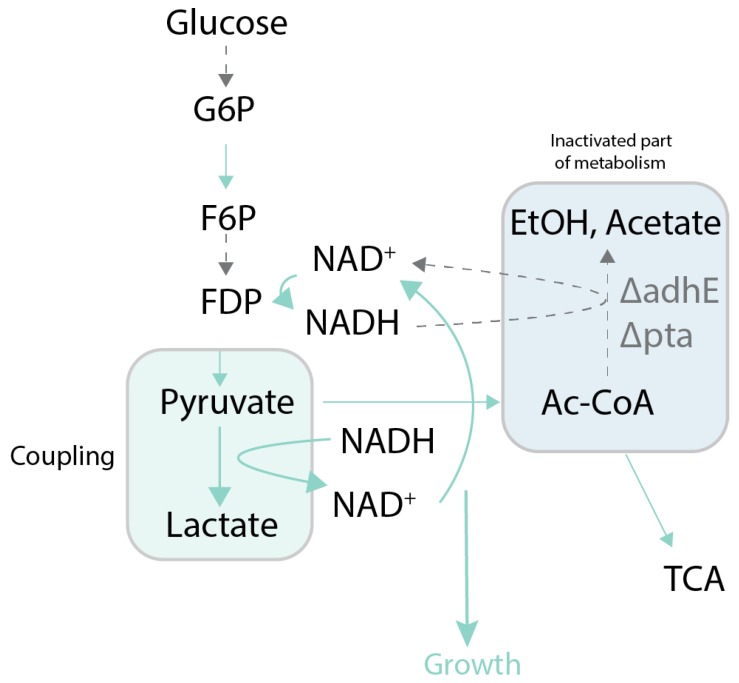
Case study of growth coupling to produce lactate [56]**.** Selection is created by deletion of fermentation pathway genes making lactate production the sole source of NADH oxidation. Green arrows and boxes represent active pathways, dotted arrows and blue boxes represent inactive parts of metabolism and pathways. adhE*:* aldehyde-alcohol dehydrogenase; pta*:* phosphate acetyltransferase; G6P: glucose 6-phosphate; F6P: fructose 6-phosphate; FDP: fructose 1,6-bisphosphate; EtOH: ethanol; TCA: citric acid cycle; Ac-CoA: acetyl coenzyme A.

One of the most commonly used growth-coupling strategies is to make the target pathway the main or only way to recycle an essential cofactor, such as NAD^+^. These redox-coupling approaches usually require anaerobic or oxygen-limited conditions, and that the product is relatively close to central carbon metabolism. For these reasons, most of the examples of redox coupling are for metabolites derived through short pathways from pyruvate. In the study by Zhou et al. [53], the authors coupled production of d-lactic acid to cell growth, and later swapped the gene for d-lactate dehydrogenase to l-lactate dehydrogenase, enabling production of l-lactic acid. In follow up studies [54,55], the authors created a strain that was able to utilize sucrose to produce d-lactic acid using the lactic acid production pathway as the sole source of NADH oxidation [56]. Fong et al. [57] coupled lactic acid production to growth by eliminating all other fermentation products, and demonstrated the ability to evolve higher yield lactic acid producers (Figure 4). A similar approach has been applied to the production of other pyruvate-derived products, including l-alanine [58] and 1-butanol [59]. In continuation with this approach, [60] proposed a method based on the same idea for production of linear higher alcohols.

**Figure 5:**
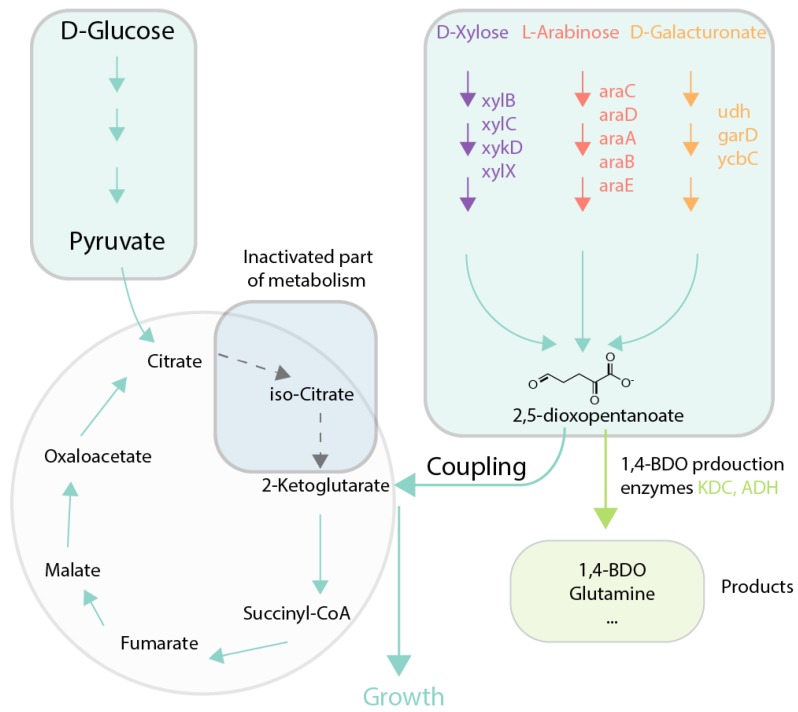
Case study of growth coupling for 1,4-BDO production [67]**.** Selection is created via disruption of tricarboxylic acid (TCA) cycle via *icd* gene deletion and introduction of nonphosphorylative metabolism as alternative for 2-ketoglutaric acid production that offers growth advantage for cells. 2,5-dioxopentanoate can be used later for production of 1,4-BDO. Green arrows and boxes are active pathways, dotted arrows and blue boxes are inactive pathways. ADH: alcohol dehydrogenase; KDC: 2-ketoacid decarboxylase; Icd*:* isocitrate dehydrogenase; 1,4-BDO: 1,4-butanediol.

Growth coupling through redox balancing can be used to ensure production of tricarboxylic acid (TCA) cycle intermediates and products derived from the TCA cycle. Growth coupling has been demonstrated for succinate and malate production [61], despite challenges associated with removing all major competing reactions which can oxidize NADH. In this study, production of malate was also coupled to ATP production, increasing effectiveness of the proposed solution. In recent work [62], the authors created an *E. coli* strain producing succinate from glycerol via deletion of the genes *adhE*, *pykAF*, *gldA*, and *pflB*. The resulting mutant did not exhibit the predicted yield, but ALE was used to improve the succinate yield. Growth coupling of succinate production was also demonstrated in [64], where a 60-fold improvement in biomass coupled succinate production and 20-fold improvement in succinate titer, compared to the reference strain, were achieved. Tai et al. [63] developed a selection strategy of coupling production of 2-ketoglutarate (2-KG) to growth. That allowed for the optimization of consumption and production pathways of major lignocellulosic biomass hydrolysate components, such as d-xylose and l-arabinose, which can be used to produce 2-KG. These evolved nonphosphorylative pathways can be further used to produce metabolites like 1,4-BDO (Figure 5). 

**Figure 6:**
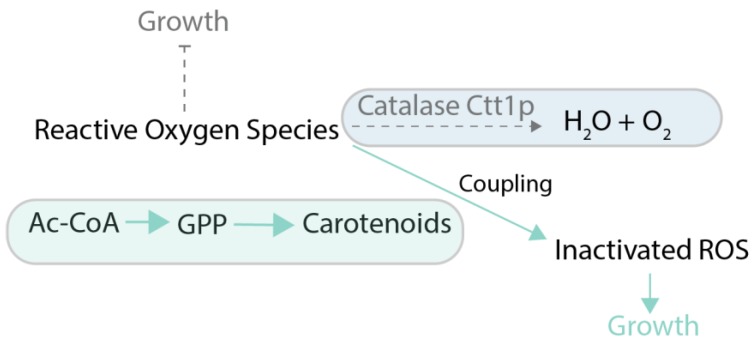
Case study of growth coupling for carotenoid production [66]**.** Selection is created via deletion of *CTT1* gene, preventing inactivation of reactive oxygen species (ROS) and addition of hydrogen peroxide (strong ROS-generating agent), inhibiting cell growth. Carotenoid production can help to inactivate ROS, thus providing a growth advantage. Ctt1p: catalase T; GPP: geranyl diphosphate; ROS: reactive oxygen species. Green arrows and boxes are active pathways, dotted arrows and blue boxes are inactive pathways.

**Figure 7:**
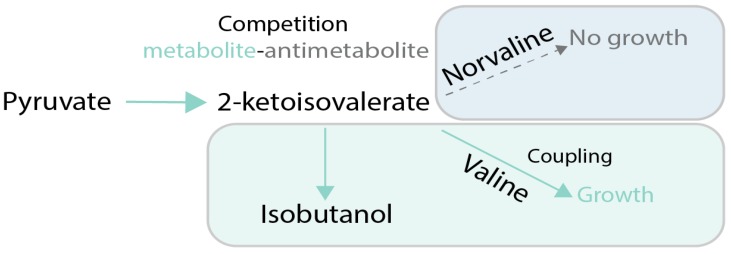
Case study of growth coupling for production of valine [61]**.** Selection is created via feeding of norvaline, a valine antimetabolite which inhibits growth. Cells can increase growth via increasing specificity and flux towards valine production. Green arrows and boxes are active pathways, dotted arrows and blue boxes are inactive pathways.

Coupling of production of a chemical to growth can also be achieved without stoichiometric coupling using stressful conditions. The idea is to employ conditions in which target metabolite alleviates the effects of the stress applied to cellular growth. One example is when the produced chemical possesses some useful activity, such as the ability to scavenge reactive oxygen species (ROS) in oxidative stress conditions. As an example of this principle, in [65], carotenoids produced in yeast were made the main source of defense against ROS by deletion of the normal ROS defense pathway (Figure 6). That allowed using evolutionary engineering to improve carotenoid production up to 3-fold compared to the parental strain. One of the classic metabolic engineering strategies is to employ toxic metabolite analogues, like norvaline, to force cells to produce the desired target metabolite (in this case, valine) in order to outcompete the toxic analogue [66] (Figure 7). The indirect coupling strategies that use various stresses as a coupling tools are more challenging to employ than stoichiometric coupling strategies, because the cells have many ways to deal with toxic compounds other than improve production of the metabolites of interest.

The examples described above aim to couple the production of a target product from a low-cost substrate, like glucose or glycerol, to growth. However, growth coupling can be more straightforwardly used to couple the activity of a single enzymatic conversion to growth. One of the simplest examples of this is the deletion of an essential enzyme, such as chorismate mutase [67], and the introduction of a library of mutagenized chorismate mutase variants. This system has been successfully used to test various synthetic proteins designs, and to evolve highly active chorismate mutase variants [68]. In the study by Smirnov et al. [69], the production of the valuable modified amino acid 4-hydroxy-l-isoleucine (4-HIL) from isoleucine was coupled to growth via “shunting” the TCA, employing l-leucine dioxygenase. Disrupting TCA with deletions of genes responsible for succinyl-CoA synthesis, this dioxygenase provides a way to bypass the succinyl-CoA step, and go directly from α-ketoglutarate to succinate, with simultaneous conversion of isoleucine to 4-HIL. This mechanism can be modified to investigate other enzymes with 2-oxoglutarate oxygenase activities [70]. Recently, the authors of this review have demonstrated a selection system that couples the production of any methylated compound to growth-enabling improvement of individual methyltransferases, as well as methyltransferase-containing pathways [71].

## 7. Challenges in Growth Selection-Based Evolutionary Engineering

Like any other method, evolutionary engineering has some limitations, especially for chemical production applications. Even though it is not difficult to set up an ALE experiment, the design of an appropriate and effective selection system is challenging, and is mostly an art, rather than a science. Most of presented case studies, except for coupling to redox balance, are developed for a single product, thus making such selection systems not generalizable. There has been significant progress in understanding how to design such selection strategies in silico, but in vivo implementation remains challenging, due to the large number of genetic changes needed. This is further compounded by phenomena such as underground metabolism or enzyme promiscuity [72], that in many cases, results in cells escaping the intended selection regime by activating a previously uncharacterized bypass pathway.

ALE is a continuous process, and exhibits challenges of diminishing returns in longer experiments [73]. In this regard, it would be beneficial to find a way to stop an ALE experiment at the right time to get most of the benefits for a minimal amount of time spent. The diminishing returns feature also makes it harder to evolve very high level production strains, even if an appropriate selection system can be designed. One possible solution to this problem is to perform ALE experiments iteratively, where genes or other genetic elements that are mutated in the previous ALE experiment are subjected to targeted diversity generation, and the next ALE is initiated with a starting population with higher diversity.

The growth-coupled ALE strategies that this review focuses on are not the only approach to perform evolutionary engineering for chemical production. Biosensors are a viable alternative that can couple almost any kind of process, including growth under, for example, antibiotic selection to the level of a metabolite [74,75]. Biosensors are a promising tool, but pose their own challenges, including the long time that it takes to develop a well-performing biosensor. This is because good biosensor systems have a large and ideally linear dynamic range, cannot be easily bypassed by mutations that alter the biosensor system itself, and are specific enough to not suffer from promiscuous activation, but also potentially usable for multiple similar chemical products. 

## 8. Conclusions

Evolutionary engineering is an established and proven method to generate microbial production strains exhibiting improvements of industrially relevant traits. Motivation to use this approach comes from both lack of complete understanding of the physiology of the host organism and elegance of using evolution as driving force to enhance features of interest. Using evolutionary engineering for chemical production is challenging due to the orthogonal nature of production of most chemicals and growth, which is usually the phenotype that is selected for in an ALE experiment. Linking production and growth can be achieved with genetic or environmental manipulations, and has been proven to allow rapid strain improvement in specific cases. The challenge for the future is to generalize these growth-coupling strategies to be applied to more products, without the need to laboriously implement specialized selection systems for each product separately. When more of these general systems are successfully developed, the complete evolutionary engineering workflow outlined in this review, including the ever-improving genetic diversity generation and omics characterization technologies, can be put to use to develop future sustainable chemical production strains.

## Figures and Tables

**Table 1:** Applications of growth-coupled selection for chemical production in microorganisms. All the coupling strategies described in this table were applied in *Escherichia coli*, except for the studies by Otero and Reyes, which were done in *Saccharomyces cerevisiae*. Some data were not available (N/A). All experimental setups used serial passaging to perform adaptive laboratory evolution (ALE).

Product	Mechanism of Coupling	Outcome	Citation	Genetic Design	Experimental setup
Prephenate	Restoring of amino acids production via production of prephenate	N/A; Evolved strain	(Kast et al., 1996) [53]	Deletions: *pheA, tyrA, aroF*	M9 medium
				Insertions: *aroH* from *Bacillus subtilis*, *tyrA* from *Erwinia herbicola*, *pheC* from *Pseudomonas aeruginosa*	
l-Lactic acid	Redox balance	Yield 93%–95% on glucose and xylose; Evolved strain	(Zhou et al., 2003) [54]	Deletion: *focA*, *pflB*, *frdB*, *frdC*, *adhE*, *ackA*	M9 medium
				Insertion: *ldhA*	
d-Lactic acid	Redox balance	Yield 88%–95% from sugar substrates; Evolved strain	(Zhou et al., 2005) [55]	Deletions: *frdA*, *pflB*, *adhE*, *ackA*	LB medium
d-Lactic acid	Redox balance	0.87 g/g glucose; Evolved strain	(Fong et al., 2005) [56]	Deletions: *adhE*, *pta*, *pfk*, *glk*	M9 medium
l-Lactic acid	Redox balance	>95% theoretical mass yield from glucose; Evolved strain	(Grabar et al., 2006) [57]	Deletions: *frdA*, *pflB*, *adhE*, *ackA*, *ldhA*, *mgsA*	NBS medium
l-Alanine	Redox balance	95% mass yield from glucose; Evolved strain	(Zhang et al., 2007) [58]	Deletions: *frdA*, *pflB*, *adhE*, *ackA*, *ldhA*, *mgsA*	NBS medium
Succinate and malate	Redox balance	Succinate 0.78 g/g glucose yield Malate 1.0 g/g glucose yield; Evolved strains	(Jantama et al., 2008) [59]	Deletions (succinate): *ldhA*, *adhE*, *ackA*, *focA*, *pflB, mgsA*, *poxB*	NBS medium
				Deletions (malate): *ldhA*, *adhE*, *ackA*, *focA*, *pflB*, *mgsA*	
1-butanol	Redox balance	70%–88% of maximum theoretical yield; Identification of mutation in Ter protein	(Shen et al., 2011) [60]	Deletions: *adhE*, *ldhA*, *frd*, *pta*	LB medium
				Insertions: *ter* from *Treponema denticola*, *atoB* from *E. coli*	
Isobutanol	Feeding with norvaline thus increasing production of valine	0.3 g/g glucose (76% of maximum theoretical yield); Set of mutations	(Smith and Liao 2011) [61]	Random mutagenesis + selection	M9 medium with norvaline
Higher-chain alcohols	Redox balance	Yield - N/A; Set of mutations	(Machado et al., 2012) [62]	Deletions: *adhE*, *ldhA*, *frd*	LB medium
				Insertions: *atoB* from *E. coli*, *adhE2*, *crt* from *C. acetobutylicum*, *hbd* from *Ralstonia eutropha*	
d-Lactic acid	Redox balance	Product yield of 85%; Evolved strain	(Wang et al., 2012) [63]	Deletions: *adhE*, *frdA*, *frdB*, *frdC*, *frdD*, *pta*, *pflB*, *aldA*, *cscR*	NBS medium
Succinate	Production of glycine and serine for biomass coupled to succinate production	0.02 g succinate/g glucose; Evolved strain	(Otero et al., 2013) [64]	Deletions: *sdh*, *ser3*, *ser33*	Minimal chemically defined medium
4-hydroxy-l-isoleucine (4-HIL)	Production of 4-HIL due to “shunting”of the citric acid cycle by simultaneously oxidizing isoleucine and α-ketoglutarate	N/A	(Smirnov et al., 2013) [65]	Deletions: *sucAB*, *aceAK*	N/A
				Insertion: *ido* from *Bacillus* *thuringiensis*	
Carotenoids	Carotenoids production as protection against oxidative stress	18 mg/g dry cell weight; Evolved strain	(Reyes et al., 2014) [66]	Deletions: CTT1	Yeast extract Peptone Dextrose medium
1,4-butanediol (1,4-BDO)	Production of 2-ketoglutarate (2-KG) via utilization of xylose and other compounds is the sole source of 2-KG	1,4-BDO yield of 0.37 g/g d-xylose; Evolved strains and set of mutations	(Tai et al., 2016) [67]	Deletions: *icd*, *xylA*, *yagE*, *yjhH* Insertions: *xylBCDX* from *C. crescentus*	M9 medium with xylose
				Deletions: *icd*, *araA* Insertions: *araCDABE* from *B. multivorans*	M9 medium with arabinose
				Deletions: *icd*, *uxaC*, *garL* Insertions: *udh* from *P. putida*, *garD* from *E. coli*, and *ycbC* from *Bacillus subtilis*	M9 medium with galactose
Succinate	Source of succinate on glycerol medium	0.68 g/g glucose; Set of mutations	(Tokuyama et al., 2018) [68]	Deletions: *adhE*, *pykAF*, *gldA*, *pflB*	M9 medium
